# Anti-Angiogenic Agent Combined with Anti-PD-1 Immunotherapy Showed Activity in Patients With Classical Hodgkin Lymphoma Who Have Failed Immunotherapy: A Retrospective Case Report Study

**DOI:** 10.3389/fimmu.2021.727464

**Published:** 2021-11-26

**Authors:** Zheng Yan, Jialin Ma, Shuna Yao, Zhihua Yao, Haiying Wang, Junfeng Chu, Shuang Zhao, Yanyan Liu

**Affiliations:** Department of Internal Medicine, Affiliated Cancer Hospital of Zhengzhou University, Henan Cancer Hospital, Zhengzhou, China

**Keywords:** PD-1, Hodgkin lymphoma, immunotherapy, apatinib, camrelizumab, anti-angiogenic agent

## Abstract

**Background:**

PD-1/PD-L1 inhibitor immunotherapy has showed impressive activity in various cancers, especially relapsed/refractory (r/r) classical Hodgkin lymphoma (cHL). However, acquired resistance is inevitable for most patients. Sometimes severe side effects also lead to treatment termination. When immunotherapy failed, alternative treatment options are limited. In the past few years, we have used the anti-angiogenic agent apatinib and PD-1 inhibitor camrelizumab to treat cHL patients who failed prior immunotherapy. In this study, we analyzed the data of these patients.

**Patients and Methods:**

Patients with r/r cHL who had failed immunotherapy and subsequently received apatinib-camrelizumab (AC) combination therapy were included in this study. Patient data were collected from medical records and follow-up system. The efficacy and safety of AC therapy were analyzed.

**Results:**

Seven patients who failed immunotherapy were identified in our database, of which five patients acquired immunotherapy resistance and two patients experienced severe side effects. They received a combination of camrelizumab (200 mg every four weeks) and apatinib (425 mg or 250 mg per day). As of the cut-off date, these patients had received a median of 4 cycles (range, 2 - 31) of treatment. Two (2/7) patients achieved complete response, four (4/7) partial response, and one (1/7) stable disease. The median progression-free survival was 10.0 months (range, 2.0 – 27.8). Low-dose apatinib (250 mg) plus camrelizumab was well tolerated and had no unexpected side effects. Besides, no reactive cutaneous capillary endothelial proliferation was observed in AC-treated patients.

**Conclusions:**

Low dose apatinib plus camrelizumab might be a promising treatment option for r/r cHL patients who have failed immunotherapy. This combination treatment is worthy of further investigation in more patients including solid cancer patients who have failed immunotherapy.

## Introduction

Classical Hodgkin lymphoma (cHL) is generally considered a curable disease; however, approximately 10%-20% of patients will develop refractory and relapsed (r/r) disease after standard treatment. The role of programmed death-1 (PD-1) and its ligand PD-L1 in tumor immunosuppression and immune escape has recently been well studied. PD-L1 is recognized as an effective predictor of PD-1/PD-L1 inhibitor immunotherapy. Due to the frequent aberrations of chromosome 9p24.1 (1), PD-L1 is universally overexpressed in cHL. Correspondingly, several PD-1 inhibitors showed excellent activity in r/r cHL patients (2–10). However, most patients can only achieve a partial response (PR), and it is just a matter of time before progression of disease (PD) occurs in these patients. In addition, immune-related adverse events (irAEs) are a concern, although the incidence of serious adverse events (SAEs) during immunotherapy is generally low. Drug resistance and/or the presence of SAEs are the major reason for immunotherapy discontinuation. Currently, treatment options for patients who have failed immunotherapy are limited. Hence, it is of great importance to develop novel treatment approaches for these populations.

Previous studies have demonstrated that anti-angiogenic agents can sensitize various tumors to immunotherapy in immunotherapy-naïve patients (11–16). However, it is not clear whether anti-angiogenic agents can resensitize the tumors of patients who have failed immunotherapy to immunotherapy rechallenge. In the past few years, we have used a combined regimen of the anti-angiogenic agent apatinib and PD-1 inhibitor camrelizumab in r/r cHL patients who had failed previous immunotherapy. Herein, we retrospectively analyzed the data of these patients.

## Patients and Methods

### Patients

Patients were confirmed to have r/r cHL by pathologists and clinicians. They received PD-1/PD-L1 inhibitor immunotherapy, but discontinued the treatment due to the presence of PD and/or SAEs. After the immunotherapy failed, they received the combination treatment of apatinib and camrelizumab (AC). This study included patients who received at least one cycle of AC combination therapy and had complete data at baseline and after treatment. The clinical characteristics, treatment procedure, adverse events, and follow-up information of eligible patients were extracted from the medical records and follow-up system.

### Assessment

The treatment response of patients with r/r cHL was assessed according to the refinement of the Lugano Classification lymphoma response criteria in the era of immunomodulatory therapy (17). The severity of adverse events was graded based on the National Cancer Institute Common Toxicity Criteria, version 5.0.

### Statistic

Progression-free survival (PFS) was defined as the duration from the start of AC combination treatment to the appearance of PD, the date of death, the start of other treatment or the last follow-up. Overall survival (OS) was defined as the duration from the start of AC combination treatment to the date of death or the last follow-up. The cut-off date for analysis was June 12, 2021.

## Results

### Characteristics of Patients

Seven patients were included in this study. There were four males and three females, with a median age of 40 years (range, 22 - 75). All patients had no noticeable family and personal medical history, except for case 5 who had a history of hypertension > 20 years. All these patients were participants in previous clinical trials of PD-1/PD-L1 inhibitor immunotherapy (NCT03505996, NCT03155425, NCT03114683). Five patients (cases 1, 3, 4, 5, and 7) withdrew from these trials due to PD. PD was confirmed by radiographic progression twice. After the first progression, treatment was continued until the disease progressed once again. Two other patients (cases 2 and 6) stopped their trials due to grade 3 checkpoint inhibitor-related pneumonitis (CIP). These patients received a median of 12 cycles (range, 1 - 29) of PD-1/PD-L1 inhibitor immunotherapy before withdrawal from their trials and the best response for these patients was PR. Before the initiation of AC combination therapy, these patients had received a median of 4 lines (range, 3 - 8) of treatment (**Table 1** and **Supplementary File 1**). The median time from the diagnosis of cHL to the initiation of AC therapy was 3.5 years (range, 2.5 – 4.2).

**Table 1:** Profile of seven patients treated with apatinib plus camrelizumab (AC).

Patient	Gender	Age (years)	Pathologic subtype	EBER	Previous treatment regimens, number of treatment cycles	Reason for discontinuation of previous immunotherapy	Initial dose of apatinib, AE	Adjusted dose of apatinib, AE	Cycles of AC therapy and best response	PFS (month)
Case 1	F	33	NSHL	+	1 ABVD, 6 2 IGEV, 2 3 ESHAP, 2 4 VMCP, 1 5 PD-L1 inhibitor, 11	PD	425 mg, G 3 elevated liver enzymes	250 mg, none	4, PR	10.0
Case 2	F	22	NSHL	–	1 ABVD, 8 2 GemOx, 2, followed by RT 3 PD-1 inhibitor, 1 4 COPP, 1 5 Liposomal doxorubicin + paclitaxel + methyhydrazine, 2 6 BV + DHAP, 2 7 GN + decitabine, 2 8 Lenalidomide, 4 months	G 3 CIP	425 mg, G 2 hand-foot syndrome	250 mg, G 1 CIP, G 1 hypothyroidism, G1 hand-foot syndrome	31, CR	27.8
Case 3	M	40	MCHL	–	1 ABVD, 4 2 IGEV, 4, followed by RT 3 ESHAP, 4, followed by ASCT 4 PD-1 inhibitor, 29	PD	425 mg, G 2 rash	250 mg, G 1 rash	2, SD	2.0
Case 4	M	40	NSHL	–	1 ABVD, 6 2 GVD, 6 3 PD-1 inhibitor, 18	PD	250 mg, none	250 mg, none	4, PR	5.7
Case 5	M	75	MCHL	–	1 ABVD, 6 2 BEACOPP, 6 3 PD-L1 inhibitor, 12	PD	250 mg, G 3 hypertension, grade 1 fatigue and anorexia	250 mg, hypertension was well controlled by hypotensor	22, CR	21.6
Case 6	F	25	NSHL	NA	1 ABVD, 6 2 IGEV, 6 3 Gemcitabine + carboplatin, 2 4 PD-1 inhibitor, 11	G 3 CIP	250 mg, G 1 hypothyroidism	250 mg, G 1 hypothyroidism, G 2 CIP	14, PR	21.4
Case 7	M	40	NSHL	–	1 ABVD, 6 2 IGEV, 2 3 GDP, 2 4 PD-L1 inhibitor, 8 5 Decitabine + PD-1 inhibitor, 4	PD	250 mg, G 1 hand-foot syndrome	250 mg, G 1 hand-foot syndrome	4, PR	5.0

EBER, EBV-encoded RNA; AE, adverse event; PFS, progression-free survival; NSHL, nodular sclerosis classical Hodgkin lymphoma; F, female; ABVD, epirubicin, bleomycin, vincristine, and dacarbazine; IGEV, ifosphamide, gemcitabine, and vinorelbine; ESHAP, etoposide, cisplatin, cytarabine, and prednisone; VMCP, vinblastine, mitoxanthrone, and prednisone; PD, progression disease; G, grade; PR, partial response; GemOx, gemcitabine and oxaliplatin; RT, radiotherapy; COPP, cyclophosphamide, vincristine, procarbazine, and prednisone; BV, brentuximab vedotin; DHAP, cisplatin, cytarabine, and dexamethasone; MCHL, mixed cellularity classical Hodgkin lymphoma; GN, gemcitabine and vinorelbine; CIP, checkpoint inhibitor-related pneumonitis; CR, complete response; M, male; ASCT, autologous stem cell transplantation; SD, stable disease; GVD, gemcitabine, vinorelbine, and liposomal doxorubicin; BEACOPP, bleomycin, etoposide, doxorubicin, cyclophosphamide, vincristine and prednisone; NA, not available; GDP, gemcitabine, cisplatin, and dexamethasone.

### Administration Schedule and Adverse Effect

Camrelizumab was given at a fixed dose of 200 mg intravenous infusion once every 4 weeks. The initial dose of apatinib was 425 mg once a day in the first three patients (cases 1 to 3). A 4-week period was defined as one cycle. Due to AEs, after the first cycle of AC therapy, the dose of apatinib in these three patients was subsequently reduced to 250 mg per day. Grade 3 elevated liver enzymes, grade 2 hand-foot syndrome, and grade 2 skin rashes occurred in cases 1, 2, and 3, respectively. Following apatinib dose reduction, the first two AEs were resolved, and the third skin rash was relieved and remained at grade 1. During the following treatment, case 2 developed grade 1 hypothyroidism, hand-foot syndrome, and CIP. The other 4 patients (cases 4 to 7) were given 250 mg of apatinib from the beginning, and of them, case 4 did not have any AEs during the treatment; case 5, who had a history of hypertension, underwent grade 3 hypertension, grade 1 fatigue and anorexia, but the hypertension was well controlled by hypotensor; case 6 experienced grade 1 hypothyroidism and grade 2 recurrent CIP; case 7 had grade 1 hand-foot syndrome (**Table 1**). The unique dermatologic toxicity of camrelizumab, called reactive cutaneous capillary endothelial proliferation (RCCEP), did not appear in any of these patients during AC combination therapy.

### Efficacy

**Figure 1:**
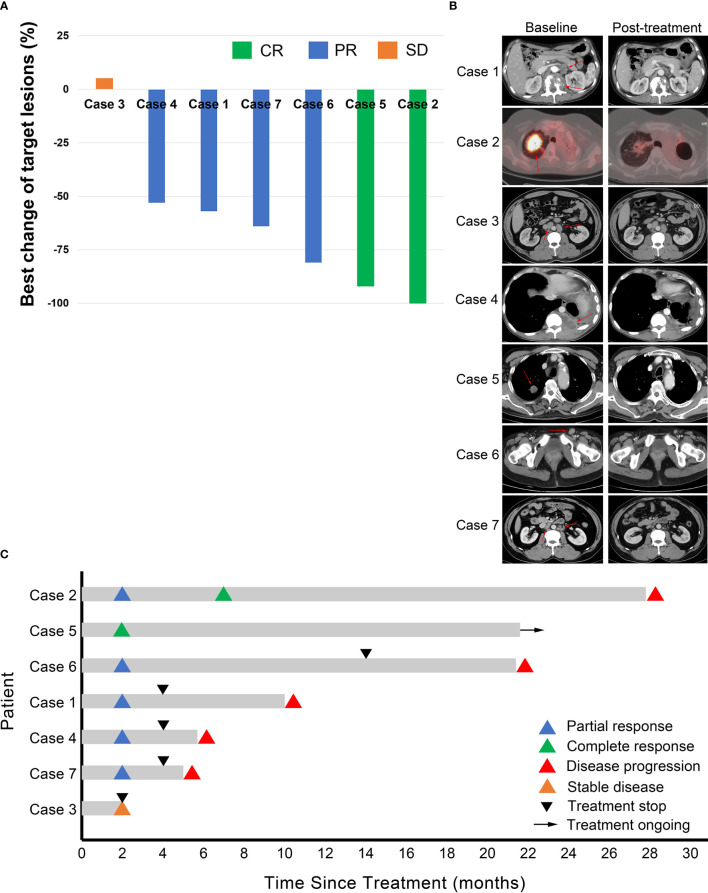
Tumor response. **(A)** Waterfall plot for best changes in tumor volume. CR, complete response; PR, partial response; SD, stable disease. **(B)** Representative images of the 7 cases at baseline and after combination treatment of apatinib and camrelizumab. Red arrows point to the tumor lesions at baseline. **(C)** Duration of responses. The length of each bar represents progression-free survival of each patient.

**Figure 2:**
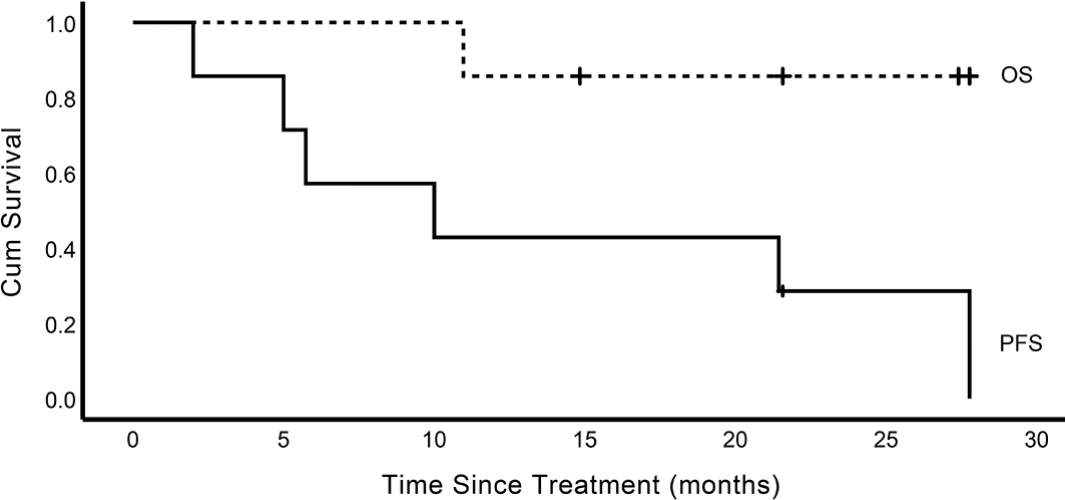
Survival curves of the 7 cases. OS, overall survival; PFS, progression-free survival.

As of the cut-off date, these patients received a median of 4 cycles (range, 2 - 31) of AC combination therapy. Two (2/7) patients (cases 2 and 5) achieved complete response (CR); four (4/7) patients (cases 1, 4, 6, and 7) achieved PR; one (1/7) patient (case 3) had stable disease (SD) (**Figure 1**). Six patients have discontinued AC therapy, of which 3 cases (cases 1, 3, and 4) discontinued due to non-progressive and non-toxic reasons, two cases (cases 2 and 7) discontinued due to disease progression, and one case (case 6) discontinued due to toxicity. Cases 1 and 4 discontinued due to financial toxicity after receiving 4 cycles of AC therapy, and then both experienced disease progression and case 4 died of lymphoma. Case 2 had a CR at the 7th month of AC therapy but relapsed at the 28th month. Case 3 discontinued the treatment at his own will when he achieved SD after two cycles of AC therapy. The PFS of this patient was 2 months according to PFS’s definition. After discontinuation of AC therapy, he received several lines of other treatments (PD-1 inhibitor combined with lenalidomide, bendamustine, or mercaptopurine). His condition remained stable for a long time but progressed in the 21st month. Case 6 discontinued treatment due to recurrent grade 2 CIP after receiving 14 cycles of AC therapy and experienced disease progression in the 22nd month. The disease of case 7 progressed after 4 cycles of treatment. Case 5 was in durable CR and received continuous treatment (**Table 1**). Tumor responses are displayed in **Figure 1** and **Supplementary File 2**. The median PFS was 10.0 months (range, 2.0 – 27.8) for these 7 patients (**Figure 2**).

## Discussion

With the ever-increasing application of immunotherapy in practice, acquired resistance to immunotherapy is becoming a major challenge in the treatment of r/r cHL and other cancers. It is urgently needed to develop strategies to overcome the resistance. The resistance mechanism of immunotherapy is very complex, involving both tumor cells and their microenvironment (TME). It has been reported that many components in TME can promote tumor cell immune evasion, including immunosuppressive cells, soluble suppressive molecules, and inhibitory receptors (18), among which vascular endothelial growth factor (VEGF)-mediated immunosuppression has been proven to play an important role in tumor escape from immune surveillance through inhibition of dendritic cell maturation, reduction of T-cell tumor infiltration, and promotion of inhibitory signaling (11, 19–27).

Anti-angiogenic agents can reprogram the tumor milieu from an immunosuppressive to an immune permissive TME by overcoming endothelial cell anergy, normalizing tumor vasculature, increasing CD4^+^ and CD8^+^ T cell infiltration, inducing adhesion molecule expression, decreasing Treg and myeloid-derived suppressor cells (MDSCs) levels, and promoting antigen presentation (28–30). In view of the immunomodulating effect of anti-angiogenic agents, they have been proposed as immunotherapy (28). Efforts have been made to incorporate anti-angiogenic agents into immunotherapy for cancer treatment. A multitude of preclinical and clinical studies have observed promising anti-tumor effects of combining anti-angiogenic agents with immune checkpoint inhibitors in many types of solid cancers, including melanoma, renal cell carcinoma, colon adenocarcinoma, gastric cancer, breast cancer, and lung cancer (12–15, 27, 31–36). Recently, several phase III studies of non-small cell lung cancer, renal cell carcinoma, and hepatocellular carcinoma have shown that the combination of anti-angiogenic agents and immunotherapy significantly improved clinical outcomes compared to their respective standards of care (27). At present, the combination of antiangiogenic agents with immunotherapy is being extensively tested in > 80 ongoing clinical trials (28). In these studies, efforts were mainly focused on immunotherapy-naïve patients. It is unclear whether the combination can be used in patients whose immunotherapy has failed.

Camrelizumab (SHR-1210) is a humanized high-affinity IgG4 monoclonal antibody against PD-1, which has been approved for used in multiple cancers in China. Apatinib is a small molecule tyrosine kinase inhibitor of vascular endothelial growth factor receptor-2 (VEGFR2), approved in China for the third-line treatment of advanced gastric cancer and the second line treatment of hepatocellular carcinoma. In early-stage clinical trials, the combination of apatinib and camrelizumab showed encouraging activity in immunotherapy-naïve patients with NSCLC, cervical cancer, hepatocellular carcinoma, and gastric cancer (32, 37, 38). In the present case series study, apatinib plus camrelizumab showed promising activity in 7 r/r cHL patients who had failed immunotherapy. Our results suggest that apatinib may resensitize resistant tumor cells to immunotherapy rechallenge. It is worth noting that the results are preliminary and the number of cases is limited. More data are needed to support this finding.

Several previous studies have demonstrated that compared with high-dose anti-angiogenic agents, low-dose anti-angiogenic agents combined with immunotherapy can better control tumor growth, because low-dose antiangiogenic agents have better immunomodulatory effect (12, 15, 16, 36). Besides, early-stage clinical trials have showed that low-dose apatinib (250 mg *vs* 500 mg) was better tolerated in patients with solid cancers when combined with immunotherapy (32, 38). Consistence with these studies, we demonstrated that when used in combination with camrelizumab, 250 mg low-dose apatinib was better tolerated in heavily pretreated r/r cHL patients.

The combination of low dose apatinib and camrelizumab did not produce unexpected toxicity. Hypertension, hand-foot syndrome, liver toxicity, fatigue, nausea and vomiting are common adverse effects of apatinib (39), while hypothyroidism is a common side effect of camrelizumab (10). Elevated liver enzymes in case 1, hand-foot syndrome in cases 2 and 7, hypertension, fatigue, and anorexia in case 5 should be attributed to apatinib; hypothyroidism in cases 2 and 6 might be attributed to camrelizumab. The attribution of rash in case 3 could not be determined. These adverse effects were mild and manageable. No other adverse effects of apatinib, such as proteinuria, diarrhea, and hemocytopenia, were observed in this case series, which may be attributed to the low dose used in these patients. Interestingly, RCCEP, the unique dermal toxicity of camrelizumab, was offset by the addition of apatinib. RCCEP occurs in approximately 90% of patients receiving camrelizumab monotherapy (10, 40). However, in the present study none of the patients receiving AC combination therapy developed RCCEP, including cases 2 and 4 who ever had grade 1 RCCEP in their previous clinical trials of camrelizumab monotherapy. This phenomenon was also observed in a prospective phase 2 trial published recently. This trial included 45 immunotherapy-naive patients with advanced cervical cancer who received AC combination therapy. RCCEP only occurred in 4 (8.9%) patients (37). The underlying mechanism of this phenomenon might be that RCCEP is caused by camrelizumab-associated modulation of VEGFR2, which is the target of apatinib (41).

In this case series, three cases (cases 2, 5, and 6) had relatively long PFS. Cases 2 and 6 discontinued the initial PD-1 inhibitor treatment due to irAE rather than resistance. Their favorable response to AC therapy was mainly due to the natural sensitivity of their lymphomas to immunotherapy. We are not sure whether the addition of apatinib reduced the severity of irAE, because case 2 received AC therapy until her disease progressed, but case 6 eventually terminated AC therapy due to recurrent grade 2 CIP after 14 treatment cycles. Case 5 who was resistant to PD-L1 inhibitor achieved a durable CR to AC combination therapy. In addition to the synergism between anti-angiogenic agent and immunotherapy, there may be incomplete cross-resistance between PD-1 inhibitors and PD-L1 inhibitors. Previously, Gelsomino et al. reported that a PD-1 inhibitor-resistant lung cancer patient responded to PD-L1 inhibitor (42), suggesting that individual patients may benefit from switching administration of PD-1 inhibitors and PD-L1 inhibitors. Case 7 underwent two lines of immunotherapy (PD-L1 inhibitor monotherapy, PD-1 inhibitor plus decitabine) before AC combination therapy, indicating that his tumor was highly resistant to immunotherapy. However, he still achieved a PR and 5 months of PFS benefit from AC therapy. Unfortunately, cases 1 and 4 discontinued AC therapy prematurely due to financial problems, which interfered with the observation of the durability of clinical response. Age, pathologic subtype, and EBV infection status did not seem to be related to the response to AC therapy (**Table 1**).

## Conclusion

In this study, low-dose apatinib plus camrelizumab showed promising activity in heavily pretreated r/rcHL patients who had failed PD-1/PD-L1 inhibitor immunotherapy. However, it is difficult to draw a definitive conclusion from this small sample size case series study. Given the rarity of r/r cHL, the combination regimen is worthy of further investigation in a larger number of patients, including solid cancer patients who have failed immunotherapy.

## Data Availability Statement

The original contributions presented in the study are included in the article/**Supplementary Material**. Further inquiries can be directed to the corresponding author.

## Ethics Statement

The studies involving human participants were reviewed and approved by Institutional Review Board of Affiliated Cancer Hospital of Zhengzhou University. The patients/participants provided their written informed consent to participate in this study. Written informed consent was obtained from the individual(s) for the publication of any potentially identifiable images or data included in this article.

## Author Contributions

Conception and design: ZYan, JM, and YL. Acquisition of data: ZYao, SY, HW, JC, and SZ. Analysis and interpretation of data: ZYan, JM, and ZHY. Statistical analysis: ZYan, JM, and YL. Drafting the article: ZYan and JM. Study supervision: YL. All authors contributed to the article and approved the submitted version.

## Conflict of Interest

The authors declare that the research was conducted in the absence of any commercial or financial relationships that could be construed as a potential conflict of interest.

## Publisher’s Note

All claims expressed in this article are solely those of the authors and do not necessarily represent those of their affiliated organizations, or those of the publisher, the editors and the reviewers. Any product that may be evaluated in this article, or claim that may be made by its manufacturer, is not guaranteed or endorsed by the publisher.

## References

[1] Piris MA Medeiros LJ Chang KC. Hodgkin Lymphoma: A Review of Pathological Features and Recent Advances in Pathogenesis. Pathology (2020) 52(1):154–65. doi: 10.1016/j.pathol.2019.09.005 31699300

[2] Goldkuhle M Dimaki M Gartlehner G Monsef I Dahm P Glossmann JP. Nivolumab for Adults With Hodgkin's Lymphoma (a Rapid Review Using the Software RobotReviewer). Cochrane Database Syst Rev (2018) 7:CD012556. doi: 10.1002/14651858.CD012556.pub2 30001476 PMC6513229

[3] Ansell SM Lesokhin AM Borrello I Halwani A Scott EC Gutierrez M. PD-1 Blockade With Nivolumab in Relapsed or Refractory Hodgkin's Lymphoma. N Engl J Med (2015) 372(4):311–9. doi: 10.1056/NEJMoa1411087 PMC4348009 25482239

[4] Bair SM Strelec LE Feldman TA Ahmed G Armand P Shah NN. Outcomes and Toxicities of Programmed Death-1 (PD-1) Inhibitors in Hodgkin Lymphoma Patients in the United States: A Real-World, Multicenter Retrospective Analysis. Oncologist (2019) 24(7):955–62. doi: 10.1634/theoncologist.2018-0538 PMC6656463 30568021

[5] Song Y Gao Q Zhang H Fan L Zhou J Zou D. Treatment of Relapsed or Refractory Classical Hodgkin Lymphoma With the Anti-PD-1, Tislelizumab: Results of a Phase 2, Single-Arm, Multicenter Study. Leukemia (2020) 34(2):533–42. doi: 10.1038/s41375-019-0545-2 PMC7214259 31520078

[6] Zhou H Fu X Li Q Niu T. Safety and Efficacy of Anti-PD-1 Monoclonal Antibodies in Patients With Relapsed or Refractory Lymphoma: A Meta-Analysis of Prospective Clinic Trails. Front Pharmacol (2019) 10:387. doi: 10.3389/fphar.2019.00387 31118893 PMC6504777

[7] Chen R Zinzani PL Fanale MA Armand P Johnson NA Brice P. Phase II Study of the Efficacy and Safety of Pembrolizumab for Relapsed/Refractory Classic Hodgkin Lymphoma. J Clin Oncol (2017) 35(19):2125–32. doi: 10.1200/JCO.2016.72.1316 PMC5791843 28441111

[8] Armand P Shipp MA Ribrag V Michot JM Zinzani PL Kuruvilla J. Programmed Death-1 Blockade With Pembrolizumab in Patients With Classical Hodgkin Lymphoma After Brentuximab Vedotin Failure. J Clin Oncol (2016) 34(31):3733–9. doi: 10.1200/JCO.2016.67.3467 PMC5791838 27354476

[9] Shi Y Su H Song Y Jiang W Sun X Qian W. Safety and Activity of Sintilimab in Patients With Relapsed or Refractory Classical Hodgkin Lymphoma (ORIENT-1): A Multicentre, Single-Arm, Phase 2 Trial. Lancet Haematol (2019) 6(1):e12–e9. doi: 10.1016/S2352-3026(18)30192-3 30612710

[10] Song Y Wu J Chen X Lin T Cao J Liu Y. A Single-Arm, Multicenter, Phase II Study of Camrelizumab in Relapsed or Refractory Classical Hodgkin Lymphoma. Clin Cancer Res (2019) 25(24):7363–9. doi: 10.1158/1078-0432.CCR-19-1680 31420358

[11] Chen DS Hurwitz H. Combinations of Bevacizumab With Cancer Immunotherapy. Cancer J (2018) 24(4):193–204. doi: 10.1097/PPO.0000000000000327 30119083

[12] Huang Y Yuan J Righi E Kamoun WS Ancukiewicz M Nezivar J. Vascular Normalizing Doses of Antiangiogenic Treatment Reprogram the Immunosuppressive Tumor Microenvironment and Enhance Immunotherapy. Proc Natl Acad Sci U.S.A. (2012) 109(43):17561–6. doi: 10.1073/pnas.1215397109 PMC3491458 23045683

[13] Wallin JJ Bendell JC Funke R Sznol M Korski K Jones S. Atezolizumab in Combination With Bevacizumab Enhances Antigen-Specific T-Cell Migration in Metastatic Renal Cell Carcinoma. Nat Commun (2016) 7:12624. doi: 10.1038/ncomms12624 27571927 PMC5013615

[14] Allen E Jabouille A Rivera LB Lodewijckx I Missiaen R Steri V. Combined Antiangiogenic and Anti-PD-L1 Therapy Stimulates Tumor Immunity Through HEV Formation. Sci Transl Med (2017) 9(385). doi: 10.1126/scitranslmed.aak9679 PMC5554432 28404866

[15] Zhao S Ren S Jiang T Zhu B Li X Zhao C. Low-Dose Apatinib Optimizes Tumor Microenvironment and Potentiates Antitumor Effect of PD-1/PD-L1 Blockade in Lung Cancer. Cancer Immunol Res (2019) 7(4):630–43. doi: 10.1158/2326-6066.CIR-17-0640 30755403

[16] Huang Y Goel S Duda DG Fukumura D Jain RK. Vascular Normalization as an Emerging Strategy to Enhance Cancer Immunotherapy. Cancer Res (2013) 73(10):2943–8. doi: 10.1158/0008-5472.CAN-12-4354 PMC3655127 23440426

[17] Cheson BD Ansell S Schwartz L Gordon LI Advani R Jacene HA. Refinement of the Lugano Classification Lymphoma Response Criteria in the Era of Immunomodulatory Therapy. Blood (2016) 128(21):2489–96. doi: 10.1182/blood-2016-05-718528 27574190

[18] Saleh R Elkord E. Acquired Resistance to Cancer Immunotherapy: Role of Tumor-Mediated Immunosuppression. Semin Cancer Biol (2019) 65:13–27. doi: 10.1016/j.semcancer.2019.07.017 31362073

[19] Motz GT Santoro SP Wang LP Garrabrant T Lastra RR Hagemann IS. Tumor Endothelium FasL Establishes a Selective Immune Barrier Promoting Tolerance in Tumors. Nat Med (2014) 20(6):607–15. doi: 10.1038/nm.3541 PMC4060245 24793239

[20] Terme M Pernot S Marcheteau E Sandoval F Benhamouda N Colussi O. VEGFA-VEGFR Pathway Blockade Inhibits Tumor-Induced Regulatory T-Cell Proliferation in Colorectal Cancer. Cancer Res (2013) 73(2):539–49. doi: 10.1158/0008-5472.CAN-12-2325 23108136

[21] Finke JH Rini B Ireland J Rayman P Richmond A Golshayan A. Sunitinib Reverses Type-1 Immune Suppression and Decreases T-Regulatory Cells in Renal Cell Carcinoma Patients. Clin Cancer Res (2008) 14(20):6674–82. doi: 10.1158/1078-0432.CCR-07-5212 18927310

[22] Bouzin C Brouet A De Vriese J Dewever J Feron O. Effects of Vascular Endothelial Growth Factor on the Lymphocyte-Endothelium Interactions: Identification of Caveolin-1 and Nitric Oxide as Control Points of Endothelial Cell Anergy. J Immunol (2007) 178(3):1505–11. doi: 10.4049/jimmunol.178.3.1505 17237399

[23] Laxmanan S Robertson SW Wang E Lau JS Briscoe DM Mukhopadhyay D. Vascular Endothelial Growth Factor Impairs the Functional Ability of Dendritic Cells Through Id Pathways. Biochem Biophys Res Commun (2005) 334(1):193–8. doi: 10.1016/j.bbrc.2005.06.065 PMC4495768 16002046

[24] Ohm JE Gabrilovich DI Sempowski GD Kisseleva E Parman KS Nadaf S. VEGF Inhibits T-Cell Development and may Contribute to Tumor-Induced Immune Suppression. Blood (2003) 101(12):4878–86. doi: 10.1182/blood-2002-07-1956 12586633

[25] Gabrilovich D Ishida T Oyama T Ran S Kravtsov V Nadaf S. Vascular Endothelial Growth Factor Inhibits the Development of Dendritic Cells and Dramatically Affects the Differentiation of Multiple Hematopoietic Lineages. vivo Blood (1998) 92(11):4150–66. doi: 10.1182/blood.V92.11.4150 9834220

[26] Gabrilovich DI Chen HL Girgis KR Cunningham HT Meny GM Nadaf S. Production of Vascular Endothelial Growth Factor by Human Tumors Inhibits the Functional Maturation of Dendritic Cells. Nat Med (1996) 2(10):1096–103. doi: 10.1038/nm1096-1096 8837607

[27] Hack SP Zhu AX Wang Y. Augmenting Anticancer Immunity Through Combined Targeting of Angiogenic and PD-1/PD-L1 Pathways: Challenges and Opportunities. Front Immunol (2020) 11:598877. doi: 10.3389/fimmu.2020.598877 33250900 PMC7674951

[28] Huinen ZR Huijbers EJM van Beijnum JR Nowak-Sliwinska P Griffioen AW. Anti-Angiogenic Agents - Overcoming Tumour Endothelial Cell Anergy and Improving Immunotherapy Outcomes. Nat Rev Clin Oncol (2021) 18(8):527–40. doi: 10.1038/s41571-021-00496-y 33833434

[29] Ciciola P Cascetta P Bianco C Formisano L Bianco R. Combining Immune Checkpoint Inhibitors With Anti-Angiogenic Agents. J Clin Med (2020) 9(3). doi: 10.3390/jcm9030675 PMC7141336 32138216

[30] Song Y Fu Y Xie Q Zhu B Wang J Zhang B. Anti-Angiogenic Agents in Combination With Immune Checkpoint Inhibitors: A Promising Strategy for Cancer Treatment. Front Immunol (2020) 11:1956. doi: 10.3389/fimmu.2020.01956 32983126 PMC7477085

[31] Hodi FS Lawrence D Lezcano C Wu X Zhou J Sasada T. Bevacizumab Plus Ipilimumab in Patients With Metastatic Melanoma. Cancer Immunol Res (2014) 2(7):632–42. doi: 10.1158/2326-6066.CIR-14-0053 PMC4306338 24838938

[32] Xu J Zhang Y Jia R Yue C Chang L Liu R. Anti-PD-1 Antibody SHR-1210 Combined With Apatinib for Advanced Hepatocellular Carcinoma, Gastric, or Esophagogastric Junction Cancer: An Open-Label, Dose Escalation and Expansion Study. Clin Cancer Res (2019) 25(2):515–23. doi: 10.1158/1078-0432.CCR-18-2484 30348638

[33] Motzer RJ Penkov K Haanen J Rini B Albiges L Campbell MT. Avelumab Plus Axitinib *Versus* Sunitinib for Advanced Renal-Cell Carcinoma. N Engl J Med (2019) 380(12):1103–15. doi: 10.1056/NEJMoa1816047 PMC6716603 30779531

[34] Rini BI Powles T Atkins MB Escudier B McDermott DF Suarez C. Atezolizumab Plus Bevacizumab *Versus* Sunitinib in Patients With Previously Untreated Metastatic Renal Cell Carcinoma (IMmotion151): A Multicentre, Open-Label, Phase 3, Randomised Controlled Trial. Lancet (2019) 393(10189):2404–15. doi: 10.1016/S0140-6736(19)30723-8 31079938

[35] Molife C Hess LM Cui ZL Li XI Beyrer J Mahoui M. Sequential Therapy With Ramucirumab and/or Checkpoint Inhibitors for non-Small-Cell Lung Cancer in Routine Practice. Future Oncol (2019) 15(25):2915–31. doi: 10.2217/fon-2018-0876 30793926

[36] Li Q Wang Y Jia W Deng H Li G Deng W. Low-Dose Anti-Angiogenic Therapy Sensitizes Breast Cancer to PD-1 Blockade. Clin Cancer Res (2020) 26(7):1712–24. doi: 10.1158/1078-0432.CCR-19-2179 31848190

[37] Lan C Shen J Wang Y Li J Liu Z He M. Camrelizumab Plus Apatinib in Patients With Advanced Cervical Cancer (CLAP): A Multicenter, Open-Label, Single-Arm, Phase II Trial. J Clin Oncol (2020) 38(34):4095–106. doi: 10.1200/JCO.20.01920 PMC7768345 33052760

[38] Zhou C Wang Y Zhao J Chen G Liu Z Gu K. Efficacy and Biomarker Analysis of Camrelizumab in Combination With Apatinib in Patients With Advanced Nonsquamous NSCLC Previously Treated With Chemotherapy. Clin Cancer Res (2021) 27(5):1296–304. doi: 10.1158/1078-0432.CCR-20-3136 33323401

[39] Li J Qin S Xu J Guo W Xiong J Bai Y. Apatinib for Chemotherapy-Refractory Advanced Metastatic Gastric Cancer: Results From a Randomized, Placebo-Controlled, Parallel-Arm, Phase II Trial. J Clin Oncol (2013) 31(26):3219–25. doi: 10.1200/JCO.2013.48.8585 23918952

[40] Chen X Ma L Wang X Mo H Wu D Lan B. Reactive Capillary Hemangiomas: A Novel Dermatologic Toxicity Following Anti-PD-1 Treatment With SHR-1210. Cancer Biol Med (2019) 16(1):173–81. doi: 10.20892/j.issn.2095-3941.2018.0172 PMC6528453 31119058

[41] Finlay WJJ Coleman JE Edwards JS Johnson KS. Anti-PD1 'SHR-1210' Aberrantly Targets Pro-Angiogenic Receptors and This Polyspecificity can be Ablated by Paratope Refinement. MAbs (2019) 11(1):26–44. doi: 10.1080/19420862.2018.1550321 30541416 PMC6343799

[42] Gelsomino F Di Federico A Filippini DM Dall'Olio FG Lamberti G Sperandi F. Overcoming Primary Resistance to PD-1 Inhibitor With Anti-PD-L1 Agent in Squamous-Cell NSCLC: Case Report. Clin Lung Cancer (2020) 21(2):e45–e8. doi: 10.1016/j.cllc.2019.11.011 31902695

